# Sickle Cell Anemia, the First Molecular Disease: Overview of Molecular Etiology, Pathophysiology, and Therapeutic Approaches

**DOI:** 10.1100/tsw.2008.157

**Published:** 2008-12-25

**Authors:** Martin H. Steinberg

**Affiliations:** Department of Medicine, Pediatrics, Pathology and Laboratory Medicine, Boston University School of Medicine, 72 E. Concord Street, Boston, MA 02118, USA

**Keywords:** fetal hemoglobin, α thalassemia, HbS, erythrocyte membrane, polymerization, nitric oxide, hydroxyurea

## Abstract

The root cause of sickle cell disease is a single β-globin gene mutation coding for the sickle β-hemoglobin chain. Sickle hemoglobin tetramers polymerize when deoxygenated, damaging the sickle erythrocyte. A multifaceted pathophysiology, triggered by erythrocyte injury induced by the sickle hemoglobin polymer, and encompassing more general cellular and tissue damage caused by hypoxia, oxidant damage, inflammation, abnormal intracellular interactions, and reduced nitric oxide bioavailability, sets off the events recognized clinically as sickle cell disease. This disease is a group of related disorders where sickle hemoglobin is the principal hemoglobin species. All have varying degrees of chronic hemolytic anemia, vasculopathy, vasoocclusive disease, acute and chronic organ damage, and shortened life span. Its complex pathophysiology, of which we have a reasonable understanding, provides multiple loci for potential therapeutic intervention.

### INTRODUCTION

Many reviews of sickle cell disease have been published, and entire books and scientific meeting proceedings have been devoted to this subject[1,2,3,4,5,6,7,8]. In this short review, early milestones and established knowledge are briefly summarized, and secondary sources often cited; newer insights into pathophysiology are emphasized citing primary sources. Another focus dwells on treatment and how drug therapy might be structured based on our current appreciation of pathophysiology.

### NOSOLOGY OF SICKLE CELL DISEASE

The heterozygous carrier of the sickle hemoglobin (HbS) gene, in the absence of another β-globin gene mutation, is said to have sickle cell trait. In carriers, because each cell contains only 30–40% HbS, polymer is not present under most conditions. Only in vessels of the renal medulla, where oxygen tension is low and the milieu is hypertonic and acidotic, can HbS polymer and sickled cells be seen. Sometimes, with vigorous exercise, sickle cells can be found in the venous circulation[9]. Carriers, except under exceptional circumstances, have few complications attributable to sickle cell trait and a normal life span (Table 1)[10]. As a result, sickle cell trait should not be considered a type of sickle cell disease. About 8% of African Americans carry the sickle cell trait. In some regions of Africa, more than a quarter of the population are carriers; very high gene frequencies are also found in some parts of Saudi Arabia, Greece, tribal populations of India, and in Brazil. When present in Caucasians, the sickle cell trait is usually present on an African haplotype chromosome and the result of generations of genetic admixture.

**TABLE 1**
**Complications of Sickle Cell Trait***

- Hyposthenuria, isosthenuria, hematuria, renal papillary necrosis, urinary tract infection with pregnancy, renal medullary carcinoma
- Increased risk of venous thromboembolism
- Increased risk of glaucoma after traumatic hyphema
- Increased risk of death after exertional heat illness
- Low-birth-weight infants
- Splenic infarction at altitude
- Difficulty leukodepleting donated blood

*Life span in sickle cell trait is normal. Complications are uncommon[10].

The clinical phenotype of sickle cell disease can be a result of many different genotypes (Table 2)[11,12]. Sickle cell anemia is defined as the homozygous state for the HbS mutation. Its birth frequency in the world's population depends on the prevalence of sickle cell trait. Many compound heterozygous conditions exist and some are common. For example, HbSC disease is found in about 1 in 800 and HbS with a β thalassemia mutation is present in about 1 in 1,200 African Americans. Collectively, all these genotypes — other less common genotypes, like HbSD, HbSE, and rare genotypes like HbS/Hb New York are also found — are included in the condition known as sickle cell disease[13]. The incidence of sickle cell disease and the proportions of each of its constituent genotypes vary widely among populations, and within a population, the incidence of the HbS gene can vary greatly. For example, in Saudi Arabia, the HbS gene has likely had an indigenous origin among people of the Eastern Oasis, whereas in the Western Oasis, the HbS gene is found on a typical African haplotype.

### PATHOPHYSIOLOGY

#### The Sickle Hemoglobin Mutation

The proximate cause of sickle cell disease is an A-to-T transversion in the codon for amino acid position 6 in the β-hemoglobin gene (*HBB*). Because of this mutation, a valine residue replaces the normal glutamic acid residue (glu6val) and HbS β-globin chains are substituted for normal HbA β-globin chains[14,15]. Studies of the haplotypes of the β-globin–like gene cluster suggested that the HbS mutations had five separate origins in equatorial Africa and, perhaps, the Middle East and India, and then spread throughout the world as populations were displaced by war, slave trading, and voluntary migration[16,17]. When agriculture developed and tropical rainforests were cleared 2,000–3,000 years ago,

**TABLE 2**
**Clinical and Hematological Features of Some Sickle Hemoglobinopathies***

| Genotype | PCV | Retic | MCV | HbF | $HbA_2$ | % Variant | Severity |
|---|---|---|---|---|---|---|---|
| Sickle cell anemia | 25 | 8 | 90 | 5 | 3 | >90% HbS | 4 |
| HbSC disease | 35 | 3 | 80 | 2 | 3 | 50% HbS and HbC | 2 |
| S-$\beta^0$ thalassemia | 27 | 7 | 82 | 7 | 5 | 90% HbS | 4 |
| S-$\beta^+$ thalassemia | 38 | 2 | 70 | 2 | 6 | 5%-30% HbA | 2 |
| HbSE disease | 35 | 3 | 75 | 2 | 3 | ~30% HbE | 2 |
| Sickle cell anemia-α thalassemia | 30 | 6 | 78 | 5 | 5 | >90% HbS | 3 |
| Sickle cell trait | 45 | 1 | 85 | 1 | 2 | 60% HbA, 40% HbS | 0 |

* Provided are average findings for a young adult for each particular genotype, in the absence of transfusion or hydroxyurea treatment. Findings in young children will differ. Within a genotype, results in an individual patient can vary widely. Severity of disease, rated from most severe (4) to absence of clinical events (0) includes complications related to sickle vasoocclusion and hemolysis. Retic = reticulocyte count. This is usually highest in sickle cell anemia, but less in other genotypes where hemolysis tends to be less severe.

standing pools of water permitted mosquitoes to flourish. The HbS gene became polymorphic as a result of the advantage for survival afforded the HbS heterozygote, given the selective pressure of mosquito-transmitted *Plasmodium falciparum* infection[18]. In this example of balanced polymorphism, the homozygote with sickle cell anemia had reduced fitness and seldom lived to reproduce, while the heterozygote carrier more often achieved reproductive age[19,20,21,22].

Globin, the apoprotein of hemoglobin, shelters the iron-bearing porphyrin heme ring where reversible oxygen binding occurs, and permits the molecule to operate efficiently in oxygen transport and its other physiological functions. Mutations can alter the primary amino acid sequence of globin polypeptides and sometimes result in clinically significant diseases called hemoglobinopathies; sickle cell disease is a prime example of this class of disorder. HbS polymerizes when it is deoxygenated, a property only of hemoglobin variants that have the HbS substitution. When a critical amount of HbS polymer accumulates within a sickle erythrocyte, cellular injury occurs and a sufficient number of damaged erythrocytes cause the phenotype of sickle cell disease recognized by hemolytic anemia and vasoocclusion (Fig. 1). Other hemoglobin variants like HbE and HbC are clinically recognizable, common, and can be found in compound heterozygotes with HbS. Thalassemias are also caused by globin gene mutations, but in contrast with hemoglobinopathies, thalassemia-causing mutations usually result in the reduction of expression or the lack of any expression of a globin gene. Typically, in thalassemia, the synthesis of one globin chain is reduced or absent; however, the structure of any globin produced by the thalassemia gene is normal. Thalassemias affecting β-globin chain synthesis are often present in compound heterozygotes with HbS. α Thalassemia, which is not allelic with mutations of *HBB*, is also frequently present in individuals with sickle cell disease[13,23].

### HbS Polymer

While hemolytic anemia and vasoocclusive events are found in varying degrees in all disease genotypes, some genotypes are clinically more severe than others. This is due in large part to variation in the cellular concentration of HbS and the propensity for polymer formation, which is highly dependent on HbS concentration. Following HbS deoxygenation and a delay of milliseconds to seconds, depending on the intracellular concentration of HbS, the HbS polymer appears in the sickle erythrocyte. The biophysics of HbS polymerization has been reviewed[24,25,26,27,28].

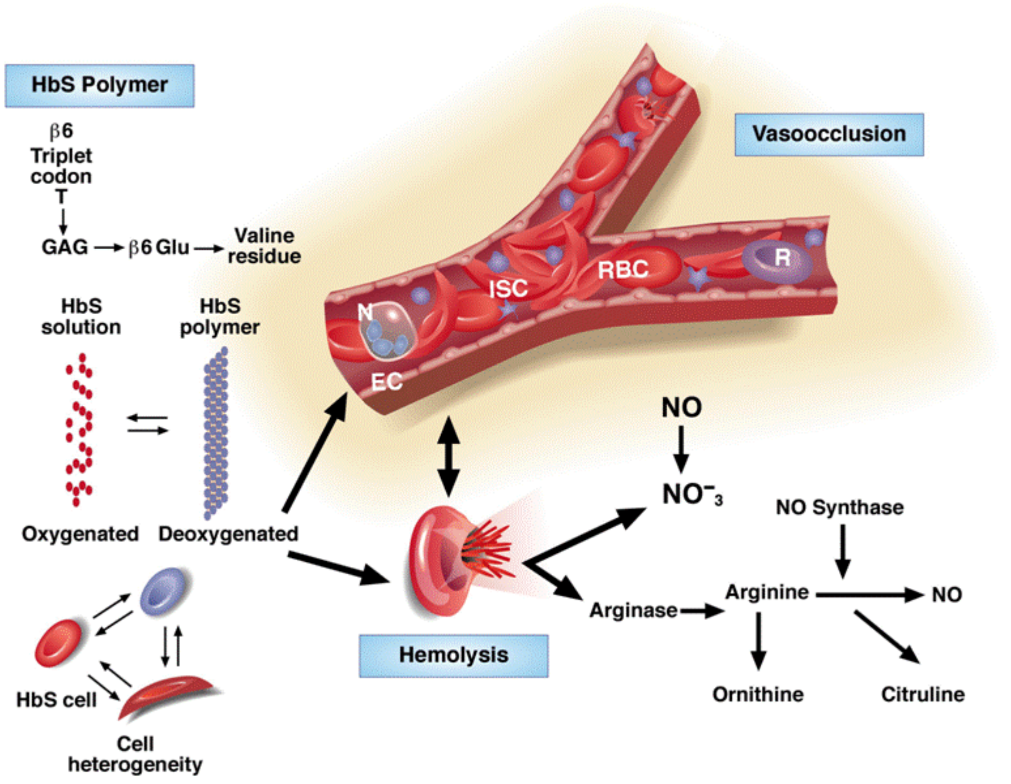

**FIGURE 1.** The pathophysiology of sickle cell disease. The HbS mutation, *HBB* glu6val, leads to β-globin chains that, when incorporated into hemoglobin tetramers with normal α-globin chains, produce a hemoglobin, HbS, which can undergo reversible polymerization when deoxygenated. The sickle polymer injures the erythrocyte and eventually produces irreversible membrane damage. These cells have a shortened life span (hemolysis), some of which occurs intravascularly consuming nitric oxide (NO). Sickle erythrocytes also lead to vasoocclusion. (From Steinberg[234].)

As sickle erythrocytes enter the microcirculation, ambient oxygen tension falls, creating molecules of deoxyHbS that have a quaternary structure that differs from oxyHbS. As the densely packed deoxyHbS molecules collide, they interact and nucleate, rapidly growing into a structured polymer, with seven pairs of elementary fibers. The HbS polymer surface provides new nucleation sites and the polymer spreads through the cell. The stable polymer decreases cell deformability, reducing their circulatory competence. If the microcirculation is successfully traversed, the return of the cell to the lungs and its exposure to high oxygen tensions allow the HbS polymer to melt. Cycles of polymerization and depolymerization ultimately cause irreversible damage to the sickle erythrocyte membrane cytoskeleton, accounting for the irreversibly sickled cells seen in the peripheral blood[29].

### Sickle Erythrocytes

The sickle hemoglobin polymer and perhaps also high concentrations of unpolymerized oxidized HbS damage the erythrocyte and its membrane[30]. Compared with normal erythrocytes, sickle erythrocytes vary in many different ways[31]. This is a result of membrane damage and the heterogeneous cellular distribution of fetal hemoglobin (HbF). HbF concentrations vary among patients with sickle cell anemia and among erythrocytes of each individual[32,33]. HbF inhibits HbS polymerization and its concentration within each cell, and distribution among all cells influences erythrocyte heterogeneity in the circulation. Cation homeostasis is impaired in some sickle cells, and as water and $K^+$ leave the cell, its density is increased. The reduced capacity of these cells to maintain their normal $K^+$ gradients is mediated by activation of cation transporters like the Gardos channel and the $K^+$:$CL^-$ cotransport channels by deoxygenation, acidification, cell swelling, $Ca^{++}$ influx, and cell sickling[34,35].

Membrane injury is also characterized by release of lipid-encased microparticles[36,37], translocation of aminophospholipids to the outer membrane leaflet[38], and damage to the membrane cytoskeleton[29]. It is the damaged sickle erythrocyte that initiates sickle vasoocclusion and hemolytic anemia.

### Sickle Vasoocclusion

The process by which normal tissue perfusion is interrupted by sickle erythrocytes is complex and incompletely understood. This process is called sickle vasoocclusion[39,40]. Obstruction might first occur in the small postcapillary venules by the entrapment of sickle cells and their adhesion to the vascular endothelium, with the ensuing lodgment of dense sickle erythrocytes, leukocytes, and platelets[41]. Nevertheless, it is not clear if this is the mechanism of vasoocclusion in all tissues. Large arteries to the brain and lungs can also become occluded, perhaps because of injury to their endothelium and activation of inflammatory pathways[42]. Experimental evidence suggests that many different elements in the sickle cell, on the sickle cell, on endothelial cells, in the subendothelial portions of the vessel wall, dissolved in the plasma, suspended in the plasma, intrinsic to the vascular wall, and extrinsic to the patient, provoke and mediate vasoocclusion[43,44,45,46,47,48,49,50]. Nevertheless, the causes of sickle vasoocclusion have not been rigorously determined and are very likely to differ temporally within an individual and differ among patients. One discussion of the vasoocclusive process has likened it to chaotic behavior[51].

Cellular damage enables adhesive interactions between sickle cells and endothelial cells[52,53,54,55]. By assorted attachment mechanisms, the association of sickle cells with endothelial cells is postulated to delay cellular passage so that polymerization, sickling, and vasoocclusion occur during microvasculature transit. Sickle cells travel in the company of leukocytes, platelets, and “stress” reticulocytes. The latter are immature erythrocytes that are found in individuals with hemolytic disease. These cells display adhesive ligands that facilitate erythrocyte-endothelial interactions[56,57,58].

### Hemolytic Anemia

The abnormal sickle erythrocyte is short lived; however, hemolytic anemia varies in intensity among the genotypes of sickle cell disease. It is most severe in patients with sickle cell anemia, less severe in individuals with sickle cell anemia and concurrent α thalassemia, and perhaps HbS-$\beta^0$ thalassemia, and least severe in patients with HbSC disease and HbS-$\beta^+$ thalassemia (Table 2). Even within a single genotype, the hemoglobin concentration is variable. For example, in sickle cell anemia, $^{51}$Cr red cell survival ranges between 2 and 21 days, and this is reflected in similarly wide variations of clinical markers of hemolysis, including total hemoglobin concentration, reticulocyte count, bilirubin level, and lactic dehydrogenase (LDH) levels[59].

Hemolysis occurs mainly extravascularly due to erythrophagocytosis by reticuloendothelial cells that recognize the damaged sickle erythrocyte[60,61]. However, red cell destruction can take place intravascularly, and the amount of intravascular hemolysis varies among patients accounting from very little to up to 30% of total hemolysis[62,63]. Chronic intravascular hemolysis ultimately leads to saturation of hemoglobin-binding proteins, allowing free hemoglobin to circulate. Plasma hemoglobin and the liberation of erythrocyte arginase into the circulation could be the driving force behind some complications of sickle cell disease because of their effect on nitric oxide (NO) bioavailability[63,64].

NO reacts rapidly with cell-free hemoglobin, producing nitrate, methemoglobin, and iron-nitrosyl hemoglobin. A normal function of NO is to activate soluble guanylate cyclase, which converts GTP to cGMP, relaxing vascular smooth muscle and causing vasodilatation (Fig. 2). The consumption of endothelial NO because of free plasma hemoglobin leads to a state of reduced endothelial NO bioavailability.

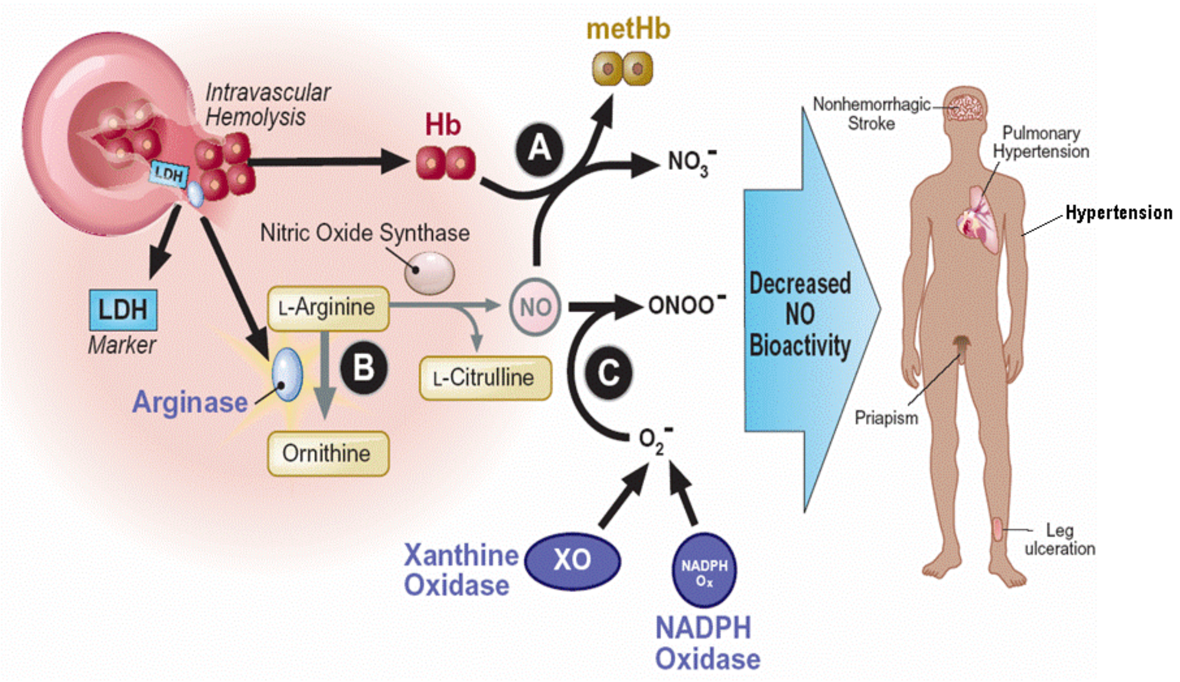

**FIGURE 2.** Intravascular hemolysis and NO bioactivity in sickle cell disease. Intravascular hemolysis releases hemoglobin, arginase, and LDH into the plasma. Hemoglobin inactivates NO, generating methemoglobin and inert nitrate. The NO synthases generate NO from the substrate L-arginine. Arginase consumes L-arginine. NO is also consumed by reactions with reactive oxygen species, producing oxygen radicals like peroxynitrite (ONOO-)(C). Decreased NO bioactivity in sickle cell disease is associated with pulmonary hypertension, priapism, leg ulceration, hypertension, and nonhemorrhagic stroke. (From Kato et al.[178].)

This impairs the downstream homeostatic vascular functions of NO, such as inhibition of platelet activation, and aggregation and transcriptional repression of the cell adhesion molecules, VCAM-1(vascular cell adhesion molecule-1), ICAM-1 (intercellular adhesion molecule-1), P-selectin, and E-selectin.

NO bioavailability is also challenged by the depletion of arginine, the substrate for the NO synthases, and by production of oxygen radicals via catalysis by hemoglobin and heme-causing superoxide-induced NO consumption. Lysed erythrocytes liberate arginase that destroys arginine, furthering endothelial NO deficiency[64,65,66,67,68,69]. For multiple reasons, hemoglobin sequestered in erythrocytes does not cause NO depletion.

The aggregate effect of reduced NO bioavailability is to inhibit normal vasodilation and cause endothelial activation and proliferation. Both hemolysis and loss of splenic function, which is a feature of sickle cell anemia, are associated with red cell membrane damage with phosphatidylserine exposure, activation of tissue factor, and thrombosis. Chronic anemia and tissue ischemia could also contribute to a proliferative vasculopathy via activation of *HIF1A* (hypoxia inducible factor)–dependent factors like *NOS2A* (inducible nitric oxide synthase), erythropoietin, and vascular endothelial growth factor.

Heme oxygenase (*HMOX1)* is an inducible enzyme that catabolizes heme and hemoglobin, and is expressed in the endothelium. In sickle transgenic mice, heme oxygenase reduces vascular inflammation and its induction inhibits reperfusion injury–induced stasis, leukocyte-endothelium interactions, and adherence molecule expression. Its inhibition increases vascular stasis. While clearly effecting vasoocclusion in sickle mice, the role of this enzyme in sickle vasoocclusion has yet to be reported in patients[70]. One might hypothesize that individuals who express the highest heme oxygenase activity, or have polymorphisms of *HMOX1* that enhance its activity, could suffer fewer or milder vasoocclusive episodes. The converse might also be true.

## CLINICAL FEATURES OF SICKLE CELL DISEASE

The following summary of the clinical features of sickle cell disease provides some highlights and recent information. Citations are limited to selected seminal papers and recent observations[6,7,71].

### Anemia

Most patients with sickle cell anemia have a moderate degree of stable anemia with a steady-state packed cell volume (PCV) between 25 and 30. Patients with HbSC disease and HbS-$\beta^+$ thalassemia are usually less anemic than individuals with sickle cell anemia and HbS-$\beta^0$ thalassemia (Table 2). Many patients with HbSC disease, or individuals with HbS-$\beta^+$ thalassemia who have high levels of HbA, especially adult men, have nearly normal PCV. A high PCV is not always a good thing because of its effects on blood viscosity. HbSC patients have a higher incidence of proliferative retinopathy, perhaps due to their increased blood viscosity, although the early loss of the peripheral retinal circulation in sickle cell anemia might prevent the later development of proliferative vascular lesions.

Advancing renal disease is a common cause of increasing chronic anemia as patients age[72]. Acutely developing severe anemia can, for reasons discussed below, occur at any age.

Sickle cell anemia patients with the most severe hemolysis can be categorized by their LDH level, assuming that liver disease and other confounding elements that might affect LDH are absent[73]. The distribution of LDH values was used as a surrogate measure of intravascular hemolysis in adults with sickle cell anemia. Chronic hyperhemolysis was defined by the top quartile of LDH level and was compared with the lowest LDH level quartile. Hyperhemolysis subjects had higher systolic blood pressure and a higher prevalence of leg ulcers, priapism, and pulmonary hypertension as defined by tricuspid regurgitant jet (TRJ) velocity or N-terminal pro brain naturietic protein (BNP) levels. Osteonecrosis and painful episodes were less prevalent in hyperhemolysis patients (Fig. 3). Hyperhemolysis was influenced by HbF level and by the presence of α thalassemia. Hyperhemolysis was also a risk factor for early death. These data suggest that serum LDH levels, when obtained at a “steady state” and which tend to remain stable over time, can identify a chronic hyperhemolysis phenotype that includes less frequent vasoocclusive pain, but earlier mortality. A number of earlier studies suggested the associations of intravascular hemolytic rate with certain disease complications[74,75,76,77,78,79,80,81]. Another clinical consequence of hemolysis is increased turnover of bile pigments and a high prevalence of gallstones.

#### *Acute Anemic Episodes*

Hemolytic anemia also places patients at risk for acutely developing severe anemia when erythropoiesis is temporarily interrupted by B19 parvovirus infection. This is the predominant cause of the aplastic crisis, typified by plummeting PCV, reticulocytopenia, and a bone marrow devoid of erythroid precursors. It is a transient process, most common in children, and often requires blood transfusion until a spontaneous recovery follows. Severe anemia can also result acutely from sequestration of blood in the spleen[82]. Rarely, if a patient’s diet is inadequate, folic acid deficiency causes megaloblastic erythropoiesis and anemia. Folic acid requirements appear to be increased because of the metabolic strain of increased erythropoiesis.

### The Early Years

Certain events complicating sickle cell disease tend to predominate in different age groups. In life’s first decades, the most common sickle cell disease–related clinical events are painful episodes, acute chest syndrome, and stroke, but all of these events can occur throughout life.

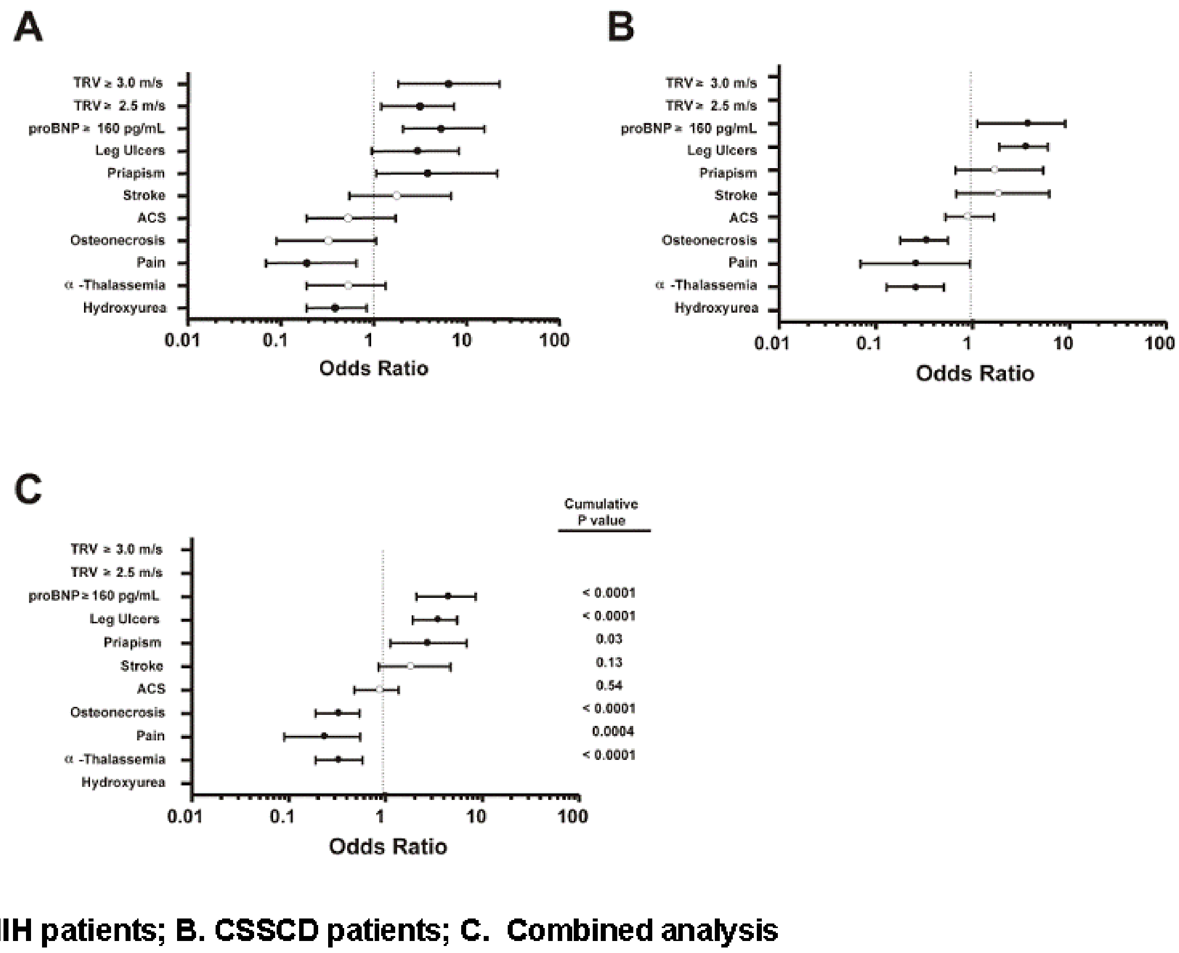

**FIGURE 3.** Clinical and laboratory findings in patients with sickle cell anemia dichotomized by LDH levels. The CSSCD group comprised 113 patients in the high LDH quartile and 111 cases in the low LDH quartile. The numbers in the NIH group were 64 and 62, respectively. (From Taylor et al.[73].)

### *The Painful Episodes*

The day-to-day management of sickle cell disease often equates with the management of acute and chronic pain. Patients manage many painful events at home so that hospital visits underestimate the frequency of pain[83]. A hospital practice overestimates the proportion of individuals who are frequent users of emergency or other acute services.

Acute painful episodes are the most commonly encountered vasoocclusive events in patients of all ages. Presumed to be caused by sickle vasoocclusion, pain often starts in young children as the hand-foot syndrome or dactylitis, a painful swelling of hands and feet due to inflammation of the metacarpal and metatarsal periosteum. Painful episodes, which last from hours to many days, usually occur with little warning and a clear precipitating event is not often found. No useful laboratory test can tell if a painful episode is occurring and the patient history is the best clue. Physical examination is usually not helpful, but sometimes there is localized swelling and pain over an involved bone. Low-grade fever and a mild increase in leukocytosis can accompany uncomplicated painful episodes, but higher temperature elevations can point to infection or extensive tissue damage.

Among patients, the number, severity, and frequency of painful episodes is quite variable[84]. On average, individuals with HbSC disease and HbS-$\beta^+$ thalassemia have half the number of painful episodes as do patients with sickle cell anemia. Patients usually know if the pain they are experiencing is different from their typical painful episode, and the wise physician should heed their advice about the need for hospitalization or likelihood that the pain has an alternative explanation. About 40% of patients do not have pain requiring a hospital visit in a given year, while 3% have more than six painful episodes per year. Having more than three painful episodes per year requiring hospitalization was associated with increased mortality in patients aged 20 years and more. HbF levels are inversely related to pain frequency; concurrent α thalassemia might increase the pain rate because of the associated decrease in hemolysis and increase in PCV[85,86,87]. Unexplained death can occur during acute painful episodes. This could be a result of an arrhythmia secondary to unrecognizable myocardial damage or perhaps a sequella of pulmonary hypertension.

A decision for hospitalization can be based on the duration and severity of the pain, and the history of prior responses to pain treatment. Excessive tachycardia, hypotension, fever beyond 101°F, leukocytosis of 20,000 cell/mm$^3$, a more than trivial fall in PCV and platelet count, hypoxia, or a new infiltrate on chest X-ray examination should prompt admission. The cornerstones of pain management, not discussed in this review, are fluid replacement and opioid analgesics[88].

### Stroke

A major complication of sickle cell anemia in early life is stroke caused by stenosis and occlusion of large vessels. Anemia causes high cerebral blood flow velocity, while sickle erythrocytes and reduced NO bioavailability damage the endothelium, causing cerebral vascular damage. Increased flow velocity can be demonstrated by transcranial Doppler ultrasonography[89,90]. Stroke is most common in patients with sickle cell anemia with much lower rates in HbSC disease and HbS-$\beta^+$ thalassemia. Subclinical neurological events and silent infarction are even more common than overt stroke. The risk of having a first stroke is 11% by age 20 years, 15% by age 30 years, and 24% by age 45 years. Cognitive abnormalities occur in sickle cell disease patients with cerebrovascular disease and stroke, and recent studies in adults suggest that neurocognitive impairment is common in adults without overt neurological impairment. The aberrant behavior of some patients is likely a result of silent infarction with deteriorating neurocognitive function.

Primary stroke prevention is possible by screening children using transcranial Doppler ultrasonography and transfusing individuals at high risk for stroke (see below).

Hemorrhagic stroke is often caused by rupture of aneurysms that might be a result of vascular injury, and these tend to happen later in life[91]. Moya moya, a proliferation of small and fragile vessels found in sickle cell patients with stenotic lesions, can also lead to cerebral hemorrhage. Hemorrhagic stroke is associated with a mortality rate of more than 20%.

### Acute Chest Syndrome

A painful episode precedes frank acute chest syndrome in 10–20% of cases. In these instances, chest syndrome develops 24–72 h after the patient seeks medical attention, suggesting that the patient should be monitored closely. Characterized by fever, chest pain, wheezing, cough, hypoxia, and a new lung infiltrate, this sometimes lethal complication affects more than half of all sickle cell anemia patients, is the second most common reason for hospitalization, and is a frequent cause of death in adults[92,93]. Occasionally, acute chest syndrome culminates with multiorgan failure.

Acute chest syndrome is frequent in children where its course is often benign; in adults, its incidence is lower, but the course tends to be more severe. Even when preoperative patients are properly prepared with blood transfusion, postoperative acute chest syndrome can develop[94]. Elevated levels of blood phospholipase $A_2$ levels have been found to be associated with acute chest syndrome and might predict its occurrence[95]. In pilot studies, transfusion of patients with pain, no obvious acute chest syndrome, and increased serum phospholipase $A_2$ levels have thwarted its development[96].

Management of acute chest syndrome is rarely targeted to the inciting cause as this is seldom apparent when treatment is most urgent. Therefore, all treatment modalities are applied to all cases, but modified according to the clinical situation estimated by respiratory distress, hypoxia, and the laboratory results. Bronchodilators, incentive spirometry, antimicrobials, and continuous or frequent monitoring of blood oxygen saturation should be used in nearly all episodes; supplemental oxygen is indicated when hypoxia is present; antibiotics are often used even if infection cannot be documented. Opioid analgesics are often needed, but their dose should be titrated carefully to avoid respiratory depression and worsening of hypoxia. Blood transfusion is the cornerstone of treatment when a patient becomes hypoxic, develops respiratory distress, has a clinically significant fall in PCV and platelet count or increase in leukocyte count, or shows any signs of multiorgan failure.

While less than 10% of all cases die, a few patients have a rapidly deteriorating course. These individuals quickly develop respiratory distress, increased oxygen requirement, extensive pulmonary opacification with effusion and edema, and can develop multiorgan failure. These individuals require ICU admission, mechanical ventilation, and corticosteroids. Excessive intravenous hydration and use of opiate analgesics could be contributing factors to the development of an ARDS-like syndrome, but it is equally likely that the nature of the triggering pulmonary event, for example, fat embolization, sets off an uncontrolled inflammatory reaction that is refractory to the usual treatment. Some patients have repeated severe acute chest syndrome. Chronic transfusion can reduce their recurrence and hydroxyurea reduces, by about half, the rate of acute chest syndrome[97]. The link between multiple episodes of acute chest syndrome and chronic pulmonary disease or pulmonary hypertension is tenuous.

#### *Other Events*

Patients with sickle cell anemia are hyposplenic early in life, and later, asplenic; they are susceptible to infection with encapsulated bacteria. Splenomegaly and splenic functions often persist in patients with HbSC disease, hence, the reduced incidence of infection and the occurrence of splenic sequestration and infarction in adults. Lasting gross splenomegaly is common in patients with sickle cell disease in Africa and the Middle East, and is related to endemic malaria. It can be associated with splenic infarction, hypersplenism, increased transfusion requirement, and may require splenectomy.

### Middle Years

#### *Pregnancy*

There is no absolute contraindication to pregnancy in sickle cell anemia and fertility is probably normal. Modern medical management generally achieves good results with pregnancy, but the rate of obstetrical complications is still higher than in a normal population. Pyelonephritis, pregnancy-induced hypertension, and caesarian section are more common, and infant birth weight was reduced compared with controls. Pregnant patients should be seen by their obstetrician every 1–2 weeks and labor closely supervised with continuous fetal monitoring. If, when, and how to use blood transfusions are the most contentious issues in the management of pregnancy. Few, if any, controlled trials in the peer-reviewed literature support the routine use of blood transfusions[98]. With good prenatal care, fetal loss and other complications are low.

#### *Surgery and Anesthesia*

Preoperative blood transfusion is generally recommended for all surgeries requiring general anesthesia. Nevertheless, the data on which this recommendation is based do not unequivocally support this practice, as a randomized untransfused comparison group was not studied[94]. In this study, simple transfusion to a

PCV of about 30 before surgery was as effective in preventing postoperative complications as exchange transfusion, and these individuals had fewer transfusion-related complications. Nevertheless, complications will still happen, including acute chest syndrome.

According to some estimates, implantable infusion ports and catheters are associated with as much as a five to ten times higher risk of complications in sickle cell anemia than when they are used in other diseases. These include thrombosis of large veins and bacteremia. Low-dose warfarin might retard thrombosis of these devices.

### Osteonecrosis and Bone Diseases

Osteonecrosis of the hip and shoulder joints affects about half of all patients with sickle cell anemia and HbSC disease. Its onset is insidious, but progressive, and most patients with early-stage disease will progress to collapse of the femoral head within 2 years. Usually, it presents with pain in and around the affected joint, at times with spasm of the surrounding muscles. It can be detected very early in its evolution by MRI, while only more advanced disease is radiographically visible[71].

Treatment with reduced weight bearing, nonsteroidal anti-inflammatory drugs, and physical therapy has been the mainstay of conservative treatment, but does not retard progression. After 3 years of follow-up, core decompression for osteonecrosis of the femoral head, a technique that percutaneously removes a core of bone in an attempt to reduce intracapital pressure, did not seem superior to physical therapy alone as a method of retarding advancing disease (ClinicalTrials.gov identifier: NCT00006130)[99]. Total hip arthroplasty can be very successful, but by 4 or 5 years after surgery, about a third of prostheses have failed.

Diffuse osteoporosis is usually present and osteomalacia due to vitamin D deficiency is very common in both children and adults, and in one study in North America, nearly all adults had reduced vitamin D levels. When vitamin D deficiency is present, treating patients with calcium and vitamin D is reasonable and appears to increase bone mineral density[100].

Osteomyelitis is often difficult to distinguish from bone infarction. Usually caused by staphylococcal infection, salmonella infection is a particular cause of sickle cell osteomyelitis.

### Leg Ulcers

Small and superficial ulcers leg ulcers heal spontaneously with rest and careful local hygiene. Nevertheless, the deep, large, painful ulcers are disabling and difficult to treat. About 5–10% of patients with sickle cell anemia aged more than 10 years had leg ulcers, but they are rare in HbSC disease or HbS-$\beta^+$ thalassemia and in young children[101]. In the tropics, leg ulcers are very common. Control of local inflammation and infection ensuring a clean granulating surface for re-epithelialization remains the mainstay of treatment. Ulcers can be excruciatingly painful and require large amounts of narcotic analgesics for relief.

### Priapism

Priapism occurs in 40% of men with sickle cell anemia. Recurrent attacks of priapism can last for several hours with tolerable discomfort and be self-limited. These episodes have been termed stuttering priapism and they usually have a nocturnal onset[102]. Major episodes of priapism often, but not always, follow a history of stuttering attacks, can last for days, and be excruciatingly painful; they often end in impotency. Priapism is a notable example of hemolysis-related sickle vasculopathy and patients also have a higher incidence of stroke, pulmonary hypertension, renal failure, and leg ulcers than individuals without priapism[73]. Conservative treatment includes analgesics, hydration, aspiration, and irrigation of the corporeal bodies. Transfusions have been used, but their effectiveness is unproven. Operative treatment includes the creation of shunts between the corpora cavernosa and spongiosum. Oral or intracavernous administration of α-adrenergic agonists might help to reverse priapism and are under study. New approaches to prevention include the use of *PDE5A* inhibitors like sildenafil[103,104,105].

### Digestive Diseases

Sickle hepatopathy, hepatic crisis, and right upper quadrant syndrome are terms applied to sickle cell–associated liver disease. Often a mélange of diverse pathologies, liver disease can be contributed to by intra- and extrahepatic cholestasis, viral hepatitis, cirrhosis, hypoxia and infarction, erythrocyte sequestration, iron overload, and drug reactions[106]. In “benign” intrahepatic cholestasis, occasionally the bilirubin level can approach 100 mg/dL. While this can resolve, progressive liver failure and death can also be the outcome. Differentiation among the potential causes of sickle cell liver disease is difficult. About 10% of patients die from liver disease[107].

### Gall Stones

Cholelithiasis, a consequence of the accelerated bile pigment turnover typical of hemolytic anemia, can appear in the first decade of life, and more than half of all adults are affected. Ultrasonography is the preferred means of their detection and laparoscopic cholecystectomy is the preferred method of dealing with symptomatic stones[108].

### “Older Patients”

Although the “middle” years might be comparatively calm compared with the emotional and physical turmoil of childhood and adolescence, beneath this illusion of disease inactivity, it is likely that sickle vasculopathy is silently progressing. With age, decades of relentless progression of sickle vasculopathy, perhaps chronic organ hypoxia, tissue microinfarction, and fibrosis claim their price in terms of organ damage and failure.

### Pulmonary Hypertension

A recently appreciated complication of sickle cell anemia is the development of pulmonary hypertension[77,109,110,111,112,113]. While included among the complications of “older” patients, it is also seen in children and in patients with HbSC disease[114,115]. Defined by a regurgitant pulmonary jet velocity of more than 2.5 m/sec by echocardiography, pulmonary hypertension is found in 30–40% of adults with sickle cell anemia. The higher frequency of pulmonary vasculopathy at autopsy study confirms the clinical impression that this is often asymptomatic or unrecognized. Occurring primarily in patients aged more than 35 years, half the patients with a regurgitant pulmonary jet velocity of more than 2.5 m/sec die in the 2 years following this diagnosis, raising the important question of why relatively low pulmonary artery pressures from a hemodynamic aspect and few symptoms are associated with such a high mortality rate?

Pulmonary hypertension is also seen in other chronic hemolytic anemias, linking its presence to hemolysis, NO bioavailability, and chronic hypoxemia. Recurrent acute chest syndrome, *in situ* thrombosis, and asplenia might also play roles. Clinically, inhaled NO has reduced pulmonary artery pressure in patients with primary pulmonary hypertension, and supplementation with the NO precursor, L-arginine, acutely reversed sickle cell pulmonary hypertension in one small study[116].

Patients with sickle cell anemia should be screened for the presence of pulmonary hypertension because of its prognostic importance. For symptomatic individuals, hydroxyurea, transfusions, sildenafil, bosentan, and epoprostenol have all been used, but controlled trials reporting the effectiveness of treatment have not been reported. How totally asymptomatic patients should be managed is unknown.

### Nephropathy

Two major abnormalities characterize the renal lesions associated with sickle cell disease, but many other renal abnormalities are found[117,118,119]. Medullary disease and hyposthenuria are present even in most carriers of sickle cell trait. Isosthenuria, distal renal tubular acidosis, and impaired potassium excretion are signs of medullary dysfunction.

Glomerular hyperfiltration, increased creatinine secretion, and very low serum creatinine is characteristic of young patients with sickle cell anemia so that renal failure can be present even with normal creatinine values. Glomerulopathy begins very early in life, but an increasing prevalence of renal failure is a hallmark of an aging population of sickle cell anemia patients. In a prospective longitudinal study of sickle cell anemia and HbSC disease, 4.2% of patients of the former and 2.4% of the latter developed renal failure with median ages of disease onset of 23 and 50 years[72,120,121]. Sixty percent of patients aged more than 40 years had proteinuria and 30% had renal insufficiency. Nephrotic syndrome was found in 40% of patients with creatinine levels above 1 mg/dL for children and 1.5 mg/dL for adults. Survival time for patients with sickle cell anemia after the diagnosis of sickle renal failure was 4 years, even when they were dialyzed, and the median age at death was 27 years.

ACE inhibitors can decrease glomerular pressure by dilating the efferent arterioles and can reduce proteinuria. In a randomized, double-blind study of 22 patients, after 6 months of 25 mg/day of captopril, a 37% reduction in microalbuminuria was found, compared with a 17% increase in placebo-treated patients[122]. Nonsteroidal anti-inflammatory drugs that inhibit the production of prostaglandins can also reduce the glomerular filtration rate in sickle cell anemia and might best be avoided in older individuals with incipient renal failure.

Screening patients for microalbuminuria might allow earlier treatment with ACE inhibitors and angiotensin receptor blockers, and forestall the development of renal failure, although a controlled clinical trial of this approach has not been reported.

### Eye Disease

Proliferative sickle retinopathy is present in more than 40% of patients with HbSC disease in the 3$^{rd}$ decade of life, but in less than 20% of individuals with sickle cell anemia. Vitreal hemorrhage and retinal detachment can lead to visual loss, but this is not common and proliferative lesions might regress spontaneously. Patients, especially those with HbSC disease, should be screened for the presence of proliferative retinopathy using flourescence angiography. The results of this examination will guide the decision of whether or not laser photocoagulation of lesions is warranted.

### Cardiovascular Complications

Cardiac exams are rarely normal; the heart is usually enlarged and the precordium hyperactive, systolic murmurs are found in most patients, and premature contractions are often present. Imaging studies have shown perfusion defects. Chest pain, a common complaint in sickle cell anemia, often leads to patients being told they have had a “heart attack”. True myocardial infarction is seldom reported, but when it is, coronary artery occlusion is uncommonly present, suggesting that small vessel disease is responsible.

Sudden unexpected and unexplained death is common in adults with sickle cell anemia and could have its origin in electrical instability.

Patients with sickle cell anemia usually have “normal” blood pressure, but this is inappropriately high when compared with controls having similar levels of anemia[123]. Survival is decreased and the risk of stroke increased as blood pressure rises, although the blood pressure at which these risks increased was below the level defining early hypertension in the normal population[124]. “Relative” hypertension in sickle cell anemia might reflect endothelial cell damage and NO scavenging[73]. Trials have not been done to guide the decision of when to begin antihypertensive treatment, what agents to use, what the blood pressure goals of treatment should be, and if blood pressure reduction can reduce the incidence of stroke or renal disease.

### TREATMENT, ESTABLISHED AND EXPERIMENTAL

#### General

Patient care is best delivered under the aegis of a multidisciplinary team led by an experienced hematologist with access to many specialists, such as orthopedic surgeons, nephrologists, and pain management experts. As a chronic disorder, attention must be paid to good nutrition and immunizations. Work and exercise as tolerated should be encouraged. Because of increased rates of red cell production and inadequate nutrition, folic acid, 1 mg daily, is often used, but this might not be necessary with a good dietary intake. Neonatal screening to detect newborns with sickle cell disease, so that they can be started on a program of prophylactic penicillin, has been shown to prevent deaths from *Streptococcus pneumoniae* infection[125,126]. Older children do not routinely need continued antibiotic prophylaxis[127].

High concentrations of inhaled oxygen cannot prevent HbS polymerization in a normoxic patient and no studies have proven the value of giving patients supplemental oxygen. When hypoxia or oxygen desaturation accompanies surgery or an acute chest syndrome, oxygen treatment can reverse these abnormalities.

Pain management is the most difficult aspect of treating sickle cell disease and useful reviews of this topic have been published[74,88,128,129,130,131,132,133,134,135,136].

In the following sections, drug treatment focused on the pathophysiology of disease is discussed with brief mention of the use of blood transfusions and stem cell transplantation. Four areas of potential treatment are discussed: agents to increase HbF and retard HbS polymerization, agents that effect inflammation and intercellular interactions, agents that help to preserve NO bioactivity, and agents that effect sickle erythrocyte density (Fig. 4).

#### Increasing HbF Concentration

HbF, whose γ-globin chains are encoded by the *HBG1* and *HBG2* genes, inhibits HbS polymerization because neither HbF ($\alpha_2\gamma_2$) nor the hybrid tetramer, $\alpha_2\gamma\beta^S$, are incorporated into the polymer phase. HbF concentrations, the distribution of HbF-containing cells, and the amount of HbF per F-cell, all vary among patients. The goal of HbF-inducing treatment is to achieve HbF levels in each sickle erythrocyte sufficient to inhibit or greatly retard HbS polymerization. Although any increment in HbF is clinically useful, based on clinical experience with the asymptomatic compound heterozygote for HbS and gene deletion hereditary persistence of HbF, at least 20% HbF in all erythrocytes should “cure” most features of disease. Unfortunately, this is unachievable with current treatment.

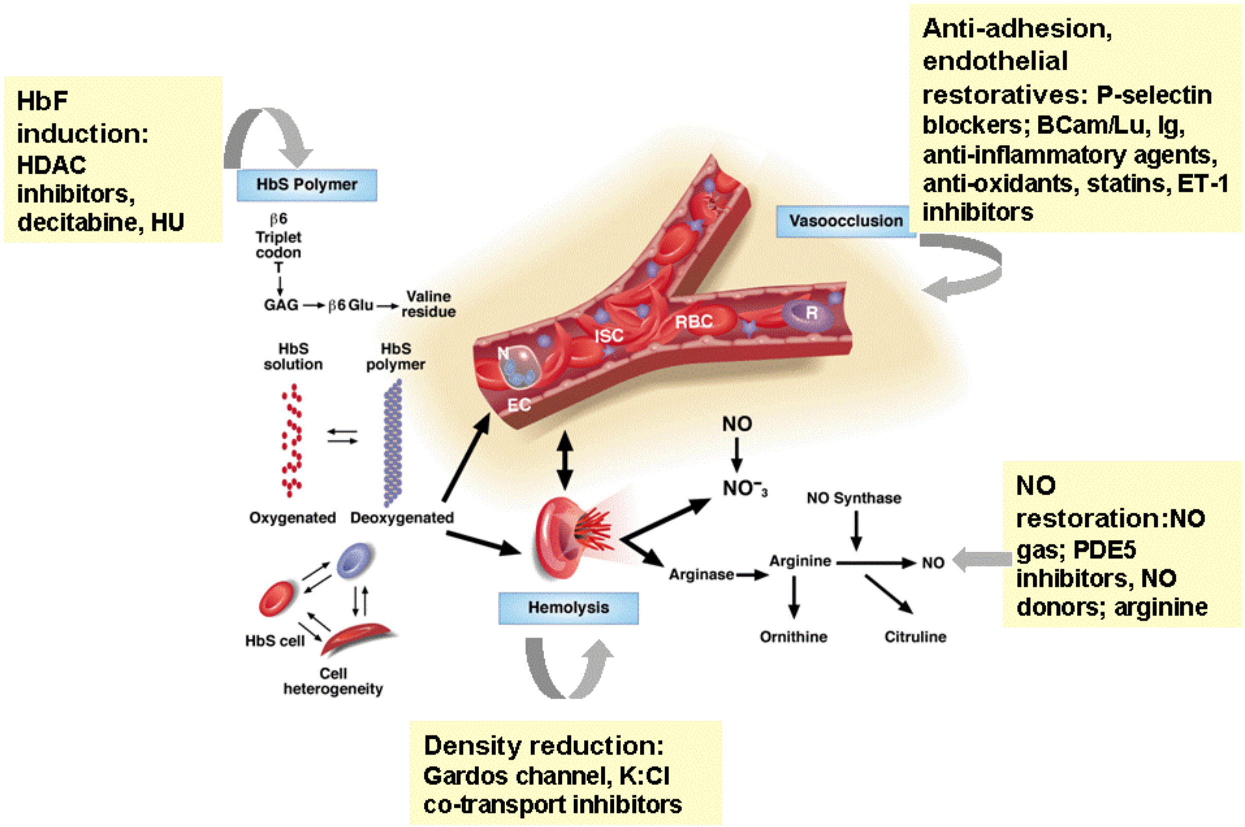

**FIGURE 4.** The pathophysiology of sickle cell disease and sites where drug treatment could be focused. Treatment by polymerization inhibition by increasing HbF concentration, interrupting intercellular interactions and reducing reperfusion injury, increasing NO bioavailability and reducing sickle erythrocyte density, provide a basis for combination chemotherapeutic approaches.

Induction of HbF has been a goal of treating sickle cell disease for decades, since the clinical and laboratory observations that HbF inhibits HbS polymerization and that higher levels of HbF are associated with reduced mortality[137,138]. Classes of HbF-inducing agents include hydroxyurea (hydroxycarbamide), decitibine, arginine butyrate, and histone deacetylase inhibitors.

### *Hydroxyurea*

Hydroxyurea, a ribonucleotide reductase inhibitor, is the sole drug with widespread regulatory approval for treating sickle cell anemia[97]. Exactly how hydroxyurea leads to a therapeutic effect is not entirely understood; however, the primary effect of this drug is likely to be due to its ability to induce high HbF levels (Fig. 5). When titrated correctly and taken faithfully, hydroxyurea can induce higher HbF levels in most patients with sickle cell anemia. Hydroxyurea is efficacious, but its effectiveness has not been realized because of poorly understood barriers to its use[139,140]. While valuable in most patients, hydroxyurea does not always help all facets of the disease, and 10–20% of patients appear biologically unable to respond to treatment; this variability might be genetically regulated[141,142,143].

In a pivotal efficacy trial in adults with sickle cell anemia, hydroxyurea reduced the frequency of hospitalization and the incidence of acute painful events, acute chest syndrome, and blood transfusion by more than 40%[97]. An observational study suggested that with moderated doses of this drug, HbF levels approached 20%[144]. In several nonrandomized studies of children titrated to either maximal doses or treated with lower doses of hydroxyurea, HbF levels approached 20%[145,146,147,148,149].

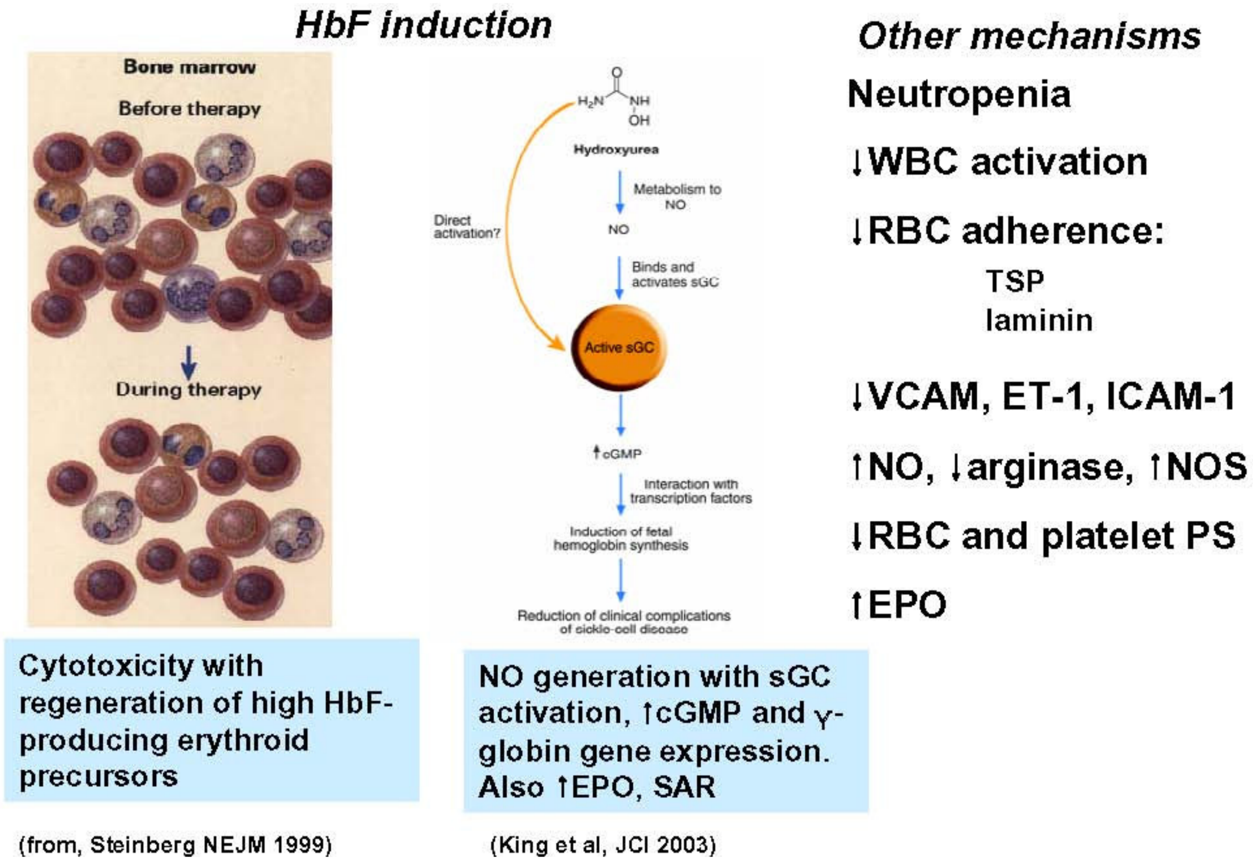

**FIGURE 5.** Mechanisms of action of hydroxyurea in sickle cell anemia[2,158]. Most evidence favors a primary effect of hydroxyurea on erythroid progenitor populations. Other mechanisms of action are possible, but some observations might be a result rather than a cause of HbF increase.

Decreased morbidity due to hydroxyurea is associated with reduced mortality. When cumulative mortality was analyzed according to total exposure to hydroxyurea, vasoocclusive complications, HbF levels and neutrophil counts, HbF levels ≥ 0.5 g/dL, absence of acute chest syndrome, and fewer painful episodes were all associated with reduced mortality[124]. Mortality was reduced 40% during 3-month intervals when patients were taking hydroxyurea, from an average of 2.6 deaths/3 months to 1.5 deaths/3 months. To date, no long-term adverse effects have been described in adults.

An ability to respond to hydroxyurea in adults could be dependent on the capacity of the marrow to withstand moderate doses of hydroxyurea, allowing erythroid precursors that synthesize HbF to regenerate preferentially[150]. Also, hydroxyurea might work by mechanisms beyond its effects on HbF. Some studies suggested that the reduction in neutrophils, monocytes, and reticulocytes accounted for the clinical benefit of hydroxyurea. Other possible mechanisms of action include effects on the sickle cell membrane, adherence molecule expression, or vascular reactivity[151,152,153,154,155]; erythrocyte cation transport; NO generation[156,157,158,159,160,161]; erythropoietin production[162]; and red cell deformability[163] (Fig. 5).

### *Demethylating Agents*

Human γ-globin genes are methylated in adult erythroid cells and hypomethylated in fetal tissue, and hypomethylation is associated with gene expression. Hypomethylation can be induced by cytidine analogs, such as 5-azacytidine, that inactivate DNA methyltransferase. This agent can induce γ-globin gene expression in primates and in patients with sickle cell anemia, but is very toxic[164]. A less toxic analog of 5-azacytidine is 5-aza-2'-deoxycytidine (decitibine). In preliminary trials, patients who failed to respond to hydroxyurea with an increase in HbF responded to decitibine, with a sustained increase of HbF that approached 14%[165]. Controlled trials of this agent in sickle cell disease have not been reported.

#### Short Chain Fatty Acids

Butyrate inhibits histone deacetylase and cyclin D1, D, and E activity, and can induce HbF expression[166]. It is also antiproliferative. Other short chain fatty acids, such as 2,2 dimethylbutyric acid and phenoxyacetic acid, can induce HbF expression, but they also stimulate cell growth, perhaps via induction of Stat 5-responsive genes; they do not inhibit histone deaceytlase[167]. Butyrates appear to modulate globin gene expression directly by binding to transcriptionally active elements, the so-called butyrate response elements, in the 5' flanking region of the γ-globin gene and also have an effect on the translational efficiency of γ-globin mRNA[166].

Short chain fatty acids that inhibit histone deacetylase might reactivate the γ-globin genes by an epigenetic mechanism. Studies have shown hyperacetylation of histones H3 and H4 at the promoters of the γ-globin genes in erythroid cells[168]. Activation of γ-globin gene expression was accompanied by suppression of β-globin gene expression, an effect that would be especially useful in sickle cell anemia as HbS production would be reduced.

#### Other Drugs that Might Inhibit HbS Polymerization

One approach to preventing HbS polymerization is to target small molecules to regions of the hemoglobin tetramer that are critical for polymer formation and stability. *In vitro* successes have been achieved with molecules as diverse as vanillin[169], oligopeptides[170], and amino acids[171]. Few new studies of this approach have been reported, perhaps because of the difficulty of toxicity, which might occur because of the necessity of achieving very high intracellular concentrations of such agents to inhibit HbS polymerization *in vivo*.

### Reducing Sickle Erythrocyte Density

Sickle erythrocytes have a reduced $Mg^+$ content, perhaps due to increased activity of the $Na^+/Mg^+$ exchanger (*KCC4*)[172]. Increasing erythrocyte $Mg^+$ *in vitro* leads to inhibition of $K^+$-$Cl^-$ cotransport and helps to preserve sickle cell hydration. Magnesium pidolate increased erythrocyte $Mg^+$ and $K^+$ content, and decreased $K^+$-$Cl^-$ cotransport in ten patients with sickle cell disease[173]. Given for 6 months in 20 patients with sickle cell disease, magnesium pidolate was associated with improved red cell hydration, and patients had a 58% reduction in the number of painful days. Randomized, double-blinded, placebo-controlled trials are evaluating this agent in sickle cell anemia and HbSC disease (NCT00143572, NCT00532883).

The Gardos channel (*KCNN4*), a calcium-activated cation transporter, is abnormally active in sickle erythrocytes. The imidazole antimycotic clotrimazole is a specific inhibitor of this channel, and its administration resulted in channel inhibition and reduction in cell dehydration[35]. ICA-17403 is a tenfold more potent blocker of the Gardos channel than clotrimazole[174]. A Phase II trial showed that this drug reduced hemolytic anemia without notable toxicity[175]. In a Phase III clinical trial, this agent also reduced hemolysis, but the trial was terminated prematurely because of the low probability of achieving its primary endpoint, a reduction in vasoocclusive events (NCT00102791).

ICA-17403 was able to reduce the hemolytic rate, probably because it reduced sickle erythrocyte density. Studies of sickle cell anemia-α thalassemia suggested that this combination, despite the reduction in cell density and hemolysis, was associated with an increased number of some disease complications dependent on blood viscosity[176]. This suggested that a drug that reduces hemolysis without effecting the intracellular hemoglobin composition might benefit some, but not all, disease complications[177]. It might be reasonable in clinical trials of agents that reduce hemolysis to craft endpoints that take into account the hemolytic complications of disease that include premature death[178,179].

### Antiadherence, Antioxidant, Anti-Inflammatory, Other Therapy

Sickle cell anemia is a vasculopathy with endothelial damage[40]. While the least developed of all therapeutic approaches, repairing or preventing sickle cell–induced vascular damage is conceptually attractive. Few clinical trials of vasoactive agents have been reported. In a Phase II study, a nonionic surfactant block copolymer, poloxamer 188, reduced the duration and speeded the resolution of acute painful episodes, an effect especially notable in children aged less than 15 years and in patients receiving hydroxyurea[180].

Other means of altering sickle cell adherence have been the subject of preliminary studies and some of these approaches are summarized.

Sulfasalazine inhibits the transcription factor, nuclear factor (NF)-κB, which modulates endothelial cell activation. This drug reduced circulating endothelial cells and endothelial expression of VCAM-1, ICAM, and E-selectin in sickle transgenic mice, while in patients, sulfasalazine reduced activation markers on circulating endothelial cells[181].

Antibodies to αVβ3 integrin, a site of sickle cell-endothelium interaction mediated by von Willebrand factor and thrombospondin, can prevent sickle cell adherence *ex vivo*[182,183].

Anionic polysaccharides, such as dextran sulfates, can inhibit sickle cell binding to endothelium via sulfated glycolipids. These molecules inhibit sickle red cell adhesion to cultured human umbilical vein endothelial cells and in *ex vivo* vessels, perhaps by preventing thrombospondin-mediated adhesion to the endothelium[184].

In a study using sickle transgenic mice, following induced inflammation by several methods, commercial intravenous immunoglobulin reduced adherent leukocytes and inhibited interactions between erythrocytes and leukocytes, causing improved microcirculatory competence and increasing survival[47,185]. While the mechanism of these effects is not known, a therapeutic trial of this agent (NCT00644865) is ongoing.

An oxygenated fluorocarbon emulsion can dislodge sickle erythrocytes after induced microvascular obstruction in an *ex vivo* rat model[186]. Lovastatin can inhibit endothelial expression of tissue factor induced by hypoxia and reperfusion injury in transgenic sickle mice, and a trials of simvastatin (NCT00508027) and atorvastatin (NCT00072826) are in progress[187].

An inhibitor of superoxide dismutase, polynitoxyl albumin, inhibited NF-κB activation and expression of VCAM-1, ICAM-1, leukocyte adherence, and vasoocclusion in sickle mice[188], effects also seen with the anti-inflammatory agent dexamethasone[189].

Intranasal cromolyn, by mechanisms unknown, has been associated with reduced erythrocyte sickling and pain intensity[190,191].

Corticosteroids have been used to reduce the duration of acute vasoocclusive episodes and sickle acute chest syndrome[192,193]. Concerns over the use of corticosteroids include immunosuppression with infection and rebound vasoocclusive events[194].

Glutamine plays an antioxidant role, preserving intracellular nicotinamide adenine dinucleotide phosphate levels that are required for glutathione recycling. Total glutathione and glutamine were lower in sickle cell disease patients than in healthy volunteers, and glutamine depletion was independently associated with pulmonary hypertension[195]. L-glutamine given orally improved the redox potential of sickle erythrocytes and the adhesion of sickle cells to human umbilical vein endothelial cells[196,197]. A

Phase II trial of this agent to evaluate its potential to reduce sickle cell acute painful episodes has been initiated (NCT00586209).

None of the diverse above-mentioned agents have been studied in carefully controlled clinical trials, and their ultimate utility in preventing disease complications remains to be seen.

Perhaps the most developed approach to interrupting adhesive interactions has focused on P-selectin inhibition. P-selectin appears to mediate the early phases of sickle-endothelial adherence and heparin can block this interaction[48,198]. A randomized, double-blind, clinical trial of 253 patients tested the safety and efficacy of a low-molecular-weight heparin, tinzaparin (Innohep®), a P-selectin blocker, over a maximum of 7 days, for the management of acute painful events[199]. Although this study had several shortcomings, compared with placebo, there was a statistically significant reduction in the number of days with the severest pain score, overall duration of painful crisis, and duration of hospitalization.

### Nitric Oxide

Several possible approaches exist to augment bioavailable NO. NO metabolites were decreased in severe sickle cell vasoocclusive crises[200]. Plasma L-arginine and serum L-arginine levels were normal in children in the steady state and fell during vasoocclusive crisis, and NO levels were normal at presentation, but decreased during hospitalization[201]. Another study found increased levels of NO derivatives in sickle cell disease.

Inhaled NO reduced pain scores and opioid use after 6 h of observation in children with acute painful episodes[202]. A Phase II clinical trial of inhaled NO used to treat acute painful episodes is ongoing (NCT00094887). Other means of increasing bioavailable NO are possible. Administration of arginine, the substrate of the NO synthases, has been associated with a reduction in pulmonary artery pressure in sickle cell anemia[116]. Among other factors, eNOS requires tetrahydrobiopterin (BH4) for optimal activity. Loss of BH4 or other cofactors leads to the uncoupling of eNOS activity from L-arginine oxidation and increased production of superoxide anion rather than NO. BH4 supplementation with sepiapterin improved eNOS function and decreased vascular endothelial cell activation[203]. A Phase II trial of this agent is in progress (NCT00445978).

*S*ildenafil is an inhibitor of phosphodiesterase 5, the enzyme that catabolizes cGMP, the intermediate that relaxes smooth muscle, to GMP[204,205]. Trials of *PDE5A* inhibitors in priapism (NCT00538564) and pulmonary hypertension (NCT0049253), two complications of sickle cell disease associated with the severity of intravascular hemolysis, have been started[104,105,206].

Nitrite, in the presence of hemoglobin acting as a deoxygenation-dependent nitrite reductase, generates NO, suggesting yet another means of therapeutically increasing NO bioavailability[207,208,209].

### Combinatorial Approaches to Treatment

Until cures of sickle cell disease by stem cell transplantation or gene therapy become widely applicable and feasible, drug treatment will remain in the forefront. A single agent that will prevent or reverse the disease pathophysiology seems an unlikely possibility. Any drug that reduces hemolysis without effecting the intracellular hemoglobin composition might benefit some, but not all, complications[176]. Multidrug combinations with available and developing agents should be the next approach in drug treatment.

Although hydroxyurea can reduce the morbidity and mortality associated with this disease in many patients[124,210], this agent does not always increase HbF to levels judged to be therapeutically adequate and even when this goal is achieved, all facets of the disease might not improve. This supports the observation that HbS polymerization might not explain the totality of disease pathophysiology and that means of treatment besides augmenting HbF production could be useful. High HbF levels appear to benefit blood viscosity complications most with a lesser effect on hemolysis-related subphenotypes[178].

These observations suggest that combinations of agents designed to increase HbF and decrease hemolysis might be more effective than either class of agent alone.

In designing clinical trials or therapeutic regimens, the first goal should be to maximize HbF levels using hydroxyurea, either alone or with other HbF-inducing agents like decitibine or a short chain fatty acid. This base of a treatment pyramid might then be built upon with agents that increase NO bioavailability, reduce intercellular interactions, reduce hemolytic anemia, and are anti-inflammatory. The pathophysiology and clinical course of sickle cell disease argues for treatment at an age as early as safely possible to forestall the progression of vasculopathy that might not be recognized clinically for many years and that culminates in irreversible organ damage. Predicting the complications and likelihood of premature mortality of disease[77,211,212] and understanding the pharmacogenomics of the different agents used should also be important components of future drug trials.

### Blood Transfusion and Iron Chelation

Transfusion of red cells can at times be lifesaving and is otherwise useful for treating some complications. Although most disease complications are likely to be greatly reduced by chronic transfusion, this approach produces iron overload, alloimmunization, loss of venous access, viral infection, and is expensive. Few controlled studies of transfusion for complications in sickle cell disease have been done and two of these were discussed above. Transfusions can prevent stroke in children deemed at high risk by estimation of their cerebral blood flow and after a stroke, transfusion appears to prevent recurrent stroke[213,214,215].

Whether exchange transfusion is preferable to simple transfusion in acute chest syndrome, stroke, or other acute complications has not been tested in clinical trials. Exchange transfusions have the benefits of being able to better control blood volume and viscosity and reduce iron accumulation, but also the drawbacks of exposure to more units of blood, difficult venous access, lack of timeliness if transfusion is urgently needed, and expense.

Anemia alone is seldom an indication for transfusion, although the association of hemolysis with many disease complications and especially pulmonary hypertension suggest that this approach should be re-evaluated. With aging and the onset of renal failure, anemia worsens and can become symptomatic. Transfusion might become necessary, although judicious use of erythropoietin can often restore the PCV to prerenal failure levels[216]. Alloimmunization occurs in about a quarter of frequently transfused patients when extended erythrocyte phenotyping has not been done[217,218]. In the presence of multiple alloantibodies, finding compatible blood is difficult.

With continued transfusion, iron overload inevitably develops and can result in heart and liver failure and multiple other complications, although these sequelae seem less common in sickle cell disease than in β thalassemia[219,220]. Serum ferritin is an inaccurate means of estimating the iron burden, and liver biopsy or perhaps MRI are more accurate means of determining tissue iron concentration and the response to chelation. Three agents are available for iron chelation: desferrioxamine, deferasirox, and deferiprone[221,222,223,224]. Desferrioxamine might be the most efficacious chelator, but its need to be given parenterally by prolonged infusion and nearly every day has limited its effectiveness in many patients. Deferasirox is said to have a similar capacity to chelate iron as desferrioxamine and can be given orally. Renal toxicity might be a limiting factor in its use. Deferiprone does not seem to as effective a chelator as the other two agents, but is orally available, selectively removes cardiac iron, and might be most effective when used with desferrioxamine or deferasirox.

### Stem Cell Transplantation

Successful stem cell transplantation can cure sickle cell anemia, but is less developed than transplantation for β thalassemia where thousands of patients have been treated[225]. Its difficulties lie in the lack of suitable marrow donors for about 90% of the eligible patients, the 5–10% mortality rate associated with the current technology of this procedure, and the poor response of patients aged more than 16 years subjected to myeloablative conditioning regimens. This has led to clinical studies of reduced-intensity conditioning with the hope of establishing stable mixed chimerism for older patients who might benefit from transplantation. However, these regimens are still in development. Patients referred for transplantation have included individuals with multiple episodes of acute chest syndrome, stroke, or severe painful episodes[226,227,228].

### Gene Therapy

Gene therapy has "cured" sickle cell disease and thalassemia in mice engineered to have these disorders[229,230]. Globin gene–containing vectors with the required regulatory and safety-einsuring elements have not been easy to design or produce. Avoiding insertional mutagenesis[231] and ensuring that there is sustained, robust, and therapeutically meaningful transgene expression at the protein level in sufficient numbers of transduced cells are problems that must be overcome[232]. In France, a Phase I/II clinical trial of gene therapy for sickle cell disease and β thalassemia using lentiviral vectors is in its very early phases, but no reports have been published[233].

### CONCLUSION

Sickle cell disease was the "first molecular disease" largely because hemoglobin was readily available from simple blood sampling, as was mRNA from reticulocytes and genomic DNA from leukocytes. This ease of sample acquisition allowed access to hemoglobin and nucleic acids that permitted finding the amino acid substitution, studies of globin gene expression, and direct detection of the mutation. Studying sickle cell disease has been instrumental in advancing our knowledge of evolution and genetics, and an increasing appreciation of its pathophysiology has permitted development of useful approaches to treatment.
